# Acromegaly: The Research and Practical Value of Noninvasive Hemodynamic Assessments *via* Impedance Cardiography

**DOI:** 10.3389/fendo.2021.793280

**Published:** 2022-01-18

**Authors:** Agnieszka Jurek, Paweł Krzesiński, Grzegorz Gielerak, Przemysław Witek, Grzegorz Zieliński, Anna Kazimierczak, Robert Wierzbowski, Małgorzata Banak, Beata Uziębło-Życzkowska

**Affiliations:** ^1^ Department of Cardiology and Internal Diseases, Military Institute of Medicine, Warsaw, Poland; ^2^ Department of Internal Medicine, Endocrinology and Diabetes, Medical University of Warsaw, Warsaw, Poland; ^3^ Department of Neurosurgery, Military Institute of Medicine, Warsaw, Poland

**Keywords:** acromegaly, impedance cardiography, cardiovascular complications, arterial, hemodynamics instability

## Abstract

**Background:**

Arterial hypertension (AH) that accompanies acromegaly (AC) may lead to cardiovascular dysfunction. Such consequences may be detected with impedance cardiography (ICG), which is a noninvasive method of hemodynamic assessment. Early detection of subclinical hemodynamic alterations in AC patients may be crucial for optimizing treatment and preventing cardiovascular remodeling. The purpose of this study was to identify the hemodynamic parameters of the cardiovascular system that differentiate patients with AC from those in the control group (CG), with a particular emphasis on potential targets for medical therapy.

**Methods:**

This observational, prospective, clinical study involved a comparative analysis of 33 AC patients with no significant comorbidities and the controls selected *via* propensity score matching based on a set of baseline characteristics (age, sex, body mass index, mean blood pressure [MBP]), with comparable proportions of AH patients. The assessed hemodynamic parameters included the stroke volume index (SI), cardiac index, systemic vascular resistance index, velocity index (VI), acceleration index, Heather index (HI), and thoracic fluid content (TFC).

**Results:**

Both the AC group and the CG had well-controlled AH (mean blood pressure of 121/77 mmHg and 119/76 mmHg, respectively). In terms of baseline characteristics, the AC group was characterized by a higher hear rate and lower creatinine levels than the CG (76.2 bpm vs. 66.8 bpm [p = 0.001] and 0.755 mg/dL vs. 0.850 mg/dL [p = 0.035], respectively). ICG assessment of AC patients and CG patients showed the former to have higher heart rates (73.5 bpm vs. 65.2 bpm; p = 0.003), lower SI (43.8 mL/m^2^ vs. 53.4 mL/m^2^; p = 0.0001), lower VI (42.1 1/1000/s vs. 49.3 1/1000/s; p = 0.037), lower HI (8.49 Ohm/s^2^ vs. 13.4 Ohm/s^2^, p ≤ 0.0001), and higher thoracic fluid content (TFC) (38.4 1/kOhm vs. 28.1 1/kOhm; p ≤ 0.0001), respectively.

**Conclusions:**

Even with well-controlled hypertension, AC is associated with a high TFC, increased heart rate, and decreased indices of cardiac contractility. Hemodynamic changes in AC patients may be detected with the modern, noninvasive diagnostic tool, ICG.

## Introduction

Acromegaly (AC) is a rare, chronic disease caused by a pituitary somatotropic adenoma secreting the growth hormone (GH). AC leads to a characteristic appearance and systemic sequelae, including structural and functional abnormalities of the cardiovascular system, which adversely affect long-term prognosis and quality of life (1, 2). AC patient mortality has been shown to be several times greater than that in the general population; however, in early diagnosed and well-controlled cases, the excess risk may be nearly completely eliminated (1–6). The hormonal misbalance associated with AC considerably affects cardiovascular function, which increases cardiovascular risk. The most common cardiovascular complications include cardiomyopathy (90%), with its consequent left ventricular (LV) dysfunction and heart failure (HF), and arterial hypertension (AH) (18%–60%) (7). AH in patients with AC is an important prognostic factor of premature death, and it accelerates the progression of cardiovascular complications (2, 8, 9). The coexistence of AH and AC exacerbates morphological and functional changes in the heart, accelerates cardiomyopathy progression, and increases the incidence of cardiomyopathy in young adults (10–12). Secondary endocrine cardiomyopathy in AC patients is caused by a long-term direct and indirect exposure of the cardiac muscle to the excessive anabolic effects of GH and insulin-like growth factor 1 (IGF-1) (13, 14). Although the resting left ventricular ejection fraction (LVEF) in patients with AC is within normal limits, a subclinical systolic LV dysfunction accompanies the diastolic LV dysfunction even at the early stages of AC and is characterized by reduced myocardial velocities and lower LV strain and strain rate values (15). Because of the many pathological effects of hormonal excess in patients with AC, most of the routine diagnostic assessments of cardiovascular hemodynamics may be of somewhat limited use, due to our incomplete understanding of AC-associated pathophysiological processes. Therefore, there is an ongoing search for novel noninvasive diagnostic methods that would help detect the abnormalities occurring early during the course of AC and increase the odds of using an optimal, targeted therapy, which would ultimately lower the cardiovascular risk.

Impedance cardiography (ICG) is a modern, noninvasive, well-established method of hemodynamic parameter assessment, which helps evaluate the cardiovascular system in terms of arterial stiffness, thoracic fluid content, cardiac pump function, etc., and may be useful in clinical evaluation of patients with AC, particularly those with coexisting AH (16–19). The use of ICG in the diagnosis of AC may help determine the individual hemodynamic status of each patient and improve our understanding of AC pathophysiology, simultaneously improving AH control and cardiovascular remodeling.

The purpose of this study was to identify those cardiovascular hemodynamic parameters that differentiate patients with AC from controls, with a particular emphasis on potential targets for medical therapy.

## Materials and Methods

### Study Population

This prospective, observational, cohort study assessed the following age-matched patient groups: 33 patients with newly diagnosed AC (mean age 47 years; 18 males; 54.5% of patients with controlled AH – the mean blood pressure [BP] of 121/77 mmHg), with no other significant comorbidities and 155 patients from the control group (CG) (mean age 47 years; 106 males; 77.4% of patients with controlled AH – the mean BP 119/76 mmHg) with no significant comorbidities.

The study was conducted in accordance with the Declaration of Helsinki and the principles of good clinical practice (GCP). The study protocol had been approved by the institutional ethics committee at the Military Institute of Medicine in Warsaw (approval No. 76/WIM/2016). A written informed consent had been obtained from each patient.

### Study Design and Patients

The AC group comprised newly diagnosed patients with clinically active acromegaly, defined based on the standard endocrine and imaging criteria in accordance with European Society of Endocrinology (ESE) guidelines: co-occurrence of the characteristic somatic features of AC and laboratory test abnormalities (the GH and IGF-1 levels above the upper limits of normal for the sex and age and a lack of GH suppression to less than 46 pmol/L (1.0 mcg/L) by a glucose load of 75 g (oral glucose tolerance test, OGTT), and a focal pituitary lesion visualized *via* magnetic resonance imaging (MRI) (20). All AC group patients underwent pituitary hormone assessments (adrenocorticotropic hormone [ACTH], follicle-stimulating hormone [FSH], luteinizing hormone [LH], thyroid stimulating hormone [TSH]) and triiodothyronine (FT3), thyroxine (FT4), cortisol and total testosterone and any preexisting or newly diagnosed carbohydrate metabolism impairment was recorded; this included type 2 diabetes mellitus (T2DM), impaired fasting glycemia (IFG), and impaired glucose tolerance (ITG). The criteria for the diagnosis of other comorbidities were applied according to the latest guidelines (21, 22).

The CG patients were the population taking part in the State-funded study “Non-invasive haemodynamic assessment in hypertension (FINE-PATH)” NCT01996085 (ClinicalTrials.gov) conducted at the *Military Institute of Medicine.* From this group, smaller subgroups, with comparable proportions of AH patients, were selected and matched for key clinical parameters [age, sex, body mass index (BMI), mean blood pressure (MBP)] for the purpose of each comparative analysis. The CG included 120 patients of either sex, with AH treated for at least 12 months and 35 healthy individuals of either sex, with no cardiovascular or other significant internal diseases, who had provided their consent to take part in this study.

Study exclusion criteria were: coronary artery disease, including a history of myocardial infarction; chronic HF with the LVEF of <50%; history of pulmonary embolism, history of a documented stroke or transient ischemic attack (TIA); chronic obstructive pulmonary disease (COPD); respiratory failure (arterial partial pressure of oxygen [PaO_2_] of < 60mmHg and/or the partial pressure of carbon dioxide [PaCO_2_] > 45mmHg); history of head injury; pregnancy; a lack of consent.

### Clinical Assessment

The clinical assessment focused on cardiovascular risk factors, including a family history of cardiovascular conditions; cardiovascular symptoms; comorbidities; nicotine dependence; impaired carbohydrate metabolism; lifestyle; office blood pressure measurement (OBPM) (systolic and diastolic BP [SBP and DBP]), heart rate (*HR)*, and BMI. The standard OBPMs were conducted with an automatic sphygmomanometer (Omron M4 Plus, Japan) in accordance with European Society of Cardiology (ESC) guidelines (21).

### Impedance Cardiography

All patients with AC underwent hemodynamic parameter assessment *via* ICG with a *Niccomo*™ device (Medis, Ilmenau, Germany). This study analysis was performed based on the ICG recordings obtained during 10-minute assessments of the following parameters measured at rest in a horizontal position: HR; SBP; DBP; cardiac pump function: cardiac output (CO [mL/min]), cardiac index (CI [L/min/m^2^]), stroke volume (SV [mL]), stroke volume index (SI [mL/m^2^]); cardiac contractility parameters: velocity index (VI [1/1000/s]: VI=1000*dZmax*Z0^−1^), reflecting the peak aortic blood flow velocity; acceleration index (ACI [1/100/s^2^]: ACI=100*dZmax*dt^−1^), reflecting the peak aortic blood acceleration; and Heather index (HI [Ohm/s^2^]: HI=dZmax*TRC), reflecting cardiac contractility. Moreover, systemic vascular resistance (SVR [dyn*s*cm^−5^], systemic vascular resistance index (SVRI [dyn*s*cm^−5^*m²]), and thoracic fluid content (TFC [1/kOhm]) were also measured. Consistent with the PREDICT study, which helped identify different risk groups based on the SI and TFC values, the cutoff values adopted in this study were < 35 mL/m^2^ and > 35 1/kOhm, respectively (23).

### Statistical Methods

Digital filing and statistical analysis of study data were conducted with *MS Office* and *Statistica* 12.0 (StatSoft Inc., Tulsa, Oklahoma, US). Continuous variables were presented as means ± standard deviation (SD), medians, and interquartile ranges; and categorical (qualitative) variables were presented as absolute values (n) and proportions (%). Continuous variable distribution was assessed visually and with the Shapiro–Wilk test. For each comparative analysis, subgroups comprising similar proportions of AH patients were selected from the CG by propensity score matching of the key clinical parameters (age, sex, BMI, MBP) that might noticeably affect the evaluated parameters. The differences between absolute values of continuous variables were analyzed with the *t*-test for normally distributed variables and the Mann–Whiney U test for non-normally distributed data. Categorical (qualitative) variables were analyzed with the chi-square and Fisher tests. Statistical significance was set at p < 0.05.

## Results

### Baseline Characteristics

All the baseline characteristics of AC and CG patients have been presented in **Table 1**. Eighteen patients (54.5%) with AC were also diagnosed with AH. In all cases, the AH had been treated with medication, usually with one or two antihypertensive agents. Out of the patients with both AC and AH, twelve patients were treated with ACEI, one patient with ARB, eight patients with CCB, six patients with beta-blockers and four patients with diuretics. T2DM was diagnosed in 6 (18.2%) of the 33 patients with AC, and prediabetes was diagnosed in 10 patients (30.3%), whereas 17 patients (51.5%) exhibited normal glucose tolerance. Out of the patients with both AC and T2DM, two patients were treated with metformin and three with metformin and insulin. Thirty-one out of the 33 patients with AC had a functioning anterior pituitary lobe. Two patients with an invasive somatotropic tumor were diagnosed with TSH deficiency; however, this was well controlled with a stable L-thyroxin dose.

**Table 1:** Baseline characteristics of acromegaly (AC) patients and control group (CG) patients.

Parameter	Mean ± SD (median; interquartile range) or n (%)
**Patents With Acromegaly (AC)**	
*Demographic Data*	
Age [years]	47.0 ± 13.5 (47.0; 38.0– 61.0)
Male sex	18 (54.5)
BMI [kg/m^2^]	27.8 ± 4.1 (27.7; 25.3–30.1)
BMI 18.5−24.9 kg/m^2^	7 (21.2)
BMI 25−29.9 kg/m^2^	17 (51.5)
BMI ≥ 30 kg/m^2^	9 (27.3)
HR [bpm]	75.6 ± 10.7 (77.0; 67.0–82.0)
SBP [mmHg]	121.0 ± 11.2 (123.0; 115.0–127.0)
SBP ≥ 140mmHg	2 (6.1)
DBP [mmHg]	77.0 ± 9.7 (77.0; 72.0–81.0)
DBP ≥ 90mmHg	2 (6.1)
*Clinical Characteristics*	
AH	18 (54.5)
T2DM	6 (18.2)
Prediabetes	10 (33.3)
Creatinine [mg/dL]	0.76 ± 0.20 (0.80; 0.60–0.80)
LVEF [%]	62.5 ± 5.1 (62.9; 60.8-66.0)
*Hormonal Profile*	
IGF-1 [ng/mL]	491.4 ± 341.8 (397.8; 281.5-527.4)
GH [ng/mL]	24.7 ± 43.0 (8.6; 4.1-24.0)
ACTH [pg/mL]	27.5 ± 14.8 (21; 16.3-34.1)
TSH [ulU/mL]	1.10 ± 0.51 (0.99; 0.82-1.54)
FT4 [pmol/L]	16.0 ± 2.9 (16.0; 13.7-17.8)
FT3 [pmol/L]	5.3 ± 1.1 (5.3; 5.0-5.8)
Total Testosterone [ng/ml]	1.58 ± 1.35 (1.59; 0.39-2.17)
Cortisol [ug/dL]	11.1 ± 6.2 (10.1; 6.0-16.8)
**Control Group (CG)**	
*Demographic Data*	
Age [years]	46.5 ± 10.0 (47.0; 40.0–53.0)
Male sex	106 (68.4)
BMI [kg/m^2^]	28.1 ± 4.0 (27.5; 25.3–30.4)
HR [bpm]	67.8 ± 9.4 (68.0; 61.0–74.0)
SBP [mmHg]	119.2 ± 10.0 (119.0; 112.0–125.0)
SBP ≥ 140 mmHg	4 (2.6)
DBP [mmHg]	76.3 ± 8.0 (76.0; 73.0–82.0)
DBP ≥ 90 mmHg	6 (3.9)
*Clinical Characteristics*	
DM	0
AH	120 (77.4)
Creatinine [mg/dL]	0.90
LVEF [%]	66.7

ACTH, adrenocorticotropic hormone; AH, arterial hypertension; BMI, body mass index; DBP, diastolic blood pressure; DM, diabetes mellitus; FSH, follicle-stimulating hormone; FT3, triiodothyronine; FT4, thyroxine; HR, heart rate; LH, luteinizing hormone; LVEF, left ventricular ejection fraction; SBP, systolic blood pressure; SD, standard deviation; TSH, thyroid stimulating hormone.

In the CG, 120 patients (77%) had AH. In all these patients, AH had been treated with medication for at least 12 months.

The evaluated ICG parameters and their values measured in the AC group have been presented in **Table 2**.

**Table 2:** Hemodynamic parameters assessed via impedance cardiography (ICG) in patients with acromegaly (AC).

Evaluated ICG Parameters	Mean ± SD (median; interquartile range) or n (%)
HR [bpm]	73.0 ± 10.6 (71.0; 65.0–80.0)
SBP [mmHg]	119.3 ± 10.1 (122.0; 115.0–124.0)
DBP [mmHg]	76.3 ± 9.2 (76.0; 72.0–80.0)
MBP [mmHg]	87.0 ± 9.5 (86.0; 80.0–91.0)
PP [mmHg]	42.9 ± 6.3 (43.0; 39.0–45.0)
SI [mL/m^2^]	44.0 ± 9.4 (43.0; 37.0–51.0)
SI < 35 mL/m^2^	6 (18.2)
CI [l/min/m^2^]	3.2 ± 0.7 (3.2; 2.7–3.6)
SVRI [dyn*s*cm^−5^*m²]	2,135 ± 578.3 (2,004; 1,761–2,387)
TAC [ml/mmHg]	1.9 ± 0.7 (1.7; 1.6–2.1)
VI [1/1000^/^s]	42.3 ± 11.5 (45.0; 35.0–50.0)
ACI [1/100/s^2^]	65.0 ± 22.5 (64.0; 50.0–79.0)
HI [Ohm/s^2^]	8.47 ± 3.20 (8.00; 6.70–11.60)
TFC [1/kOhm]	38.5 ± 6.9 (38.7; 33.8–42.4)
TFC > 35 1/kOhm	23 (69.7)

ACI, acceleration index; CI, cardiac index; DBP, diastolic blood pressure; HI, Heather index; HR, heart rate; ICG, impedance cardiography; MBP, mean blood pressure; PP, pulse pressure; SBP, systolic blood pressure; SI, stroke index; SVRI, systemic vascular resistance index; TAC, total artery compliance; TFC, thoracic fluid content; VI, velocity index.

### Comparison of AC and CG Patients in Terms of Their Baseline Characteristics and the Hemodynamic Parameters Assessed *via* ICG

A comparative analysis of the baseline characteristics in the AC group and in the CG has been presented in **Table 3**.

**Table 3:** Comparative analysis of the general characteristics of the control group (CG) and the acromegaly (AC) group.

Parameters	CG mean ± SD/n (%)	AC mean ± SD/n (%)	p-value
**General Characteristics**			
Age [years]	48.4 ± 9.5	47.5 ± 13.3	0.771
BMI [kg/m^2^]	28.7 ± 4.2	27.7 ± 4.2	0.363
HR [bpm]	66.8 ± 10.1	76.2 ± 10.4	0.001
SBP [mmHg]	121.8 ± 9.3	120.8 ± 11.4	0.693
DBP [mmHg]	78.7 ± 8.4	77.4 ± 9.5	0.579
Creatinine [mg/dL]	0.85 ± 0.15	0.76 ± 0.19	0.035
**Pharmacotherapy**			
ACEI	12	12	0.921
ARB	4	1	0.151
CCB	5	8	0.384
beta-blockers	2	6	0.143
diuretics	7	4	0.292

ACEI, angiotensin-converting-enzyme inhibitors, ARB, angiotensin receptor blockers, BMI, body mass index; CCB, calcium channel blockers, DBP, diastolic blood pressure; HR, heart rate; SBP, systolic blood pressure; SD, standard deviation.

**Figure 1:**
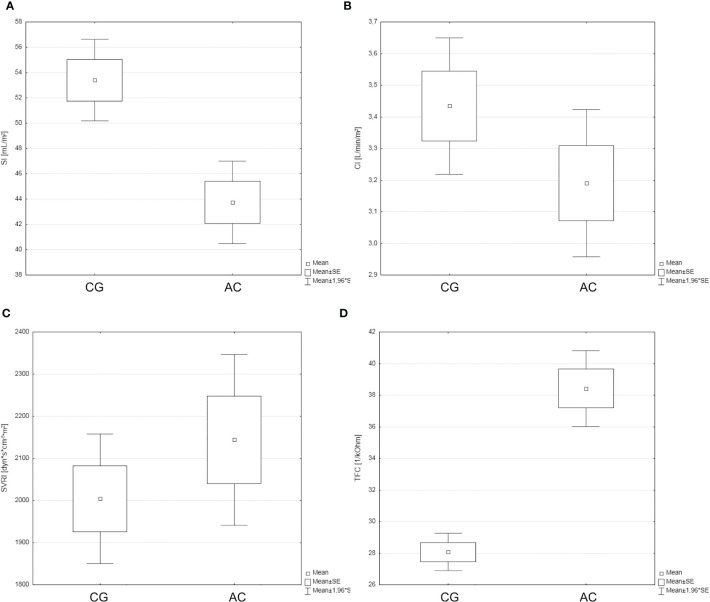
The hemodynamic profiles of the study groups [control group (CG) and acromegaly (AC) group] with a particular focus on SI **(A)**, CI **(B)**, SVRI **(C)**, and TFC **(D)**.

The analysis revealed significant differences in the HR and serum creatinine levels. Patients with AC were characterized by significantly higher HR values and significantly lower serum creatinine levels than those in the CG (HR of 76.2 bpm vs. 66.8 bpm, respectively; p=0.001; and creatinine of 0.76 mg/dL vs. 0.85 mg/dL, respectively; p=0.035). No significant differences between the groups were observed in any of the other evaluated parameters. A comparative analysis of the evaluated ICG parameters showed a number of differences between the groups (**Table 4** and **Figure 1**). In comparison with CG patients, the patients with AC were shown to have significantly higher HR values (p=0.003), significantly lower SI (p=0.0001), significantly lower indices of cardiac contractility: VI (p=0.037) and HI (p<0.0001), and significantly higher TFC (p<0.0001).

**Table 4:** Comparison of the control group (CG) and the acromegaly (AC) group in terms of the hemodynamic parameters assessed via impedance cardiography.

Parameters	CG mean ± SD	AC mean ± SD	p-value
**Impedance Cardiography**			
HR [bpm]	65.2 ± 10.8	73.5 ± 10.4	**0.003**
SBP [mmHg]	119.9 ± 10.8	119.0 ± 10.1	0.941
DBP [mmHg]	78.5 ± 8.6	76.8 ± 9.1	0.351
MBP [mmHg]	89.1 ± 9.0	87.3 ± 9.5	0.464
PP [mmHg]	41.4 ± 5.9	42.3 ± 5.1	0.327
SI [mL/m^2^]	53.4 ± 9.3	43.8 ± 9.4	**0.0001**
CI [L/min/m^2^]	3.4 ± 0.6	3.2 ± 0.7	0.138
SVRI [dyn*s*cm^−5^*m²]	2,004 ± 442.7	2,144 ± 585.3	0.285
VI [1/1000^/^s]	49.3 ± 15.2	42.1 ± 11.6	**0.037**
ACI [1/100/s^2^]	75.8 ± 33.3	64.8 ± 22.9	0.131
HI [Ohm/s^2^]	13.4 ± 4.1	8.49 ± 3.25	**<0.0001**
TFC [1/kOhm]	28.1 ± 3.4	38.4 ± 7.00	**<0.0001**

ACI, acceleration index; CI, cardiac index; DBP, diastolic blood pressure; HI, Heather index; HR, heart rate; MBP, mean blood pressure; PP, pulse pressure; SBP, systolic blood pressure; SI, stroke index; SVRI, systemic vascular resistance index; TAC, total artery compliance; TFC, thoracic fluid content; VI, velocity index.

Statistical significance was adopted at p < 0.05.

A comparative analysis of the CG and AC patients in terms of other selected parameters has been presented in **Table 5**. ICG assessments showed a low SI (< 35 mL/m^2^), which reflects impaired LV function, in 3.1% of CG patients and in 18.8% of AC patients (p=0.045). ICG assessments also showed a high TFC (> 35 1/kOhm) in 3.1% of patients from the CG and in 68.8% of patients from the AC group (p< 0.0001).

**Table 5:** Comparison of the control group (CG) and the acromegaly (AC) group in terms of selected parameters. .

Parameters	CG x/y (z%)	AC x/y (z%)	p-value
**General Characteristics**			
Male sex, n [%]	15/32 (46.9)	17/32 (53.1)	0.617
AH, n [%]	20/32 (62.5)	17/32 (53.1)	0.448
SI < 35 mL/m^2^, n [%]	1/32 (3.1)	6/32 (18.8)	**0.045**
TFC > 35 1/kOhm, n [%]	1/32 (3.1)	22/32 (68.8)	**<0.0001**

AH, arterial hypertension; SI, stroke index; TFC, thoracic fluid content. Statistical significance was adopted at p < 0.05.

There was no significant difference between AC patients with hypertension and those without hypertension for any of the evaluated hemodynamic parameters. There was also no significant difference between AC patients with and without diabetes or glucose intolerance for any of the evaluated hemodynamic parameters. No significant correlations between hemodynamic parameters and GH/IGF1 were noted (**Supplementary Table S1**).

## Discussion

Our study demonstrated cardiovascular hemodynamic abnormalities in patients newly diagnosed with AC, even despite an adequate AH control in most of those patients. Comprehensive hemodynamic assessments *via* ICG revealed that the hemodynamic profiles of AC patients are different than those in individuals with normal somatotropic axis function. Moreover, ICG is easy to use and may be used in virtually any hospital ward or outpatient clinic.

The study group comprised patients newly diagnosed with AC, without any other comorbidities that might considerably impair their cardiovascular function. The data on demographic characteristics, past medical history, and cardiovascular system function collected in this study were only somewhat similar to those reported in other clinical studies on cardiovascular dysfunction in patients with AC (2, 7–9, 24–37). The lower rates of AH, HF, and carbohydrate metabolism impairment in comparison with those reported by other authors were most likely due to the fact that those other clinical studies recruited patients with AC with baseline evidence of severe cardiovascular dysfunction and multiple comorbidities, which might have affected the final results. We would like to mention that detailed cardiovascular hemodynamic assessment *via* ICG is the first attempt to use this technique in patients with AC.

### TFC and the Mechanism of AH

The development of cardiovascular complications in patients with AC is responsible for an increases mortality due to structural and functional alterations in the cardiovascular system (36, 38). One of the most common adverse consequences of long-term tissue exposure to GH excess in patients with AC is the development of AH. It has been already shown that an altered circadian BP profile in patients with active acromegaly (non-dipping profile) may be associated with higher GH and IGF-1 levels (39). In our study, 54.5% out of the 33 patients with AC were diagnosed with AH. Importantly, AH is also an independent predictor of mortality in AC patients, with the following factors potentially contributing to the development of AH in this patient group: rapid HR, increased LV afterload, increased SV and CO, endothelial dysfunction, changes in systemic vascular resistance, and reduced coronary perfusion pressure (24, 40), with direct antidiuretic effects of GH and IGF-1 in the kidneys also playing an important role (41). Excessively high levels of these hormones may increase the renal reabsorption of sodium and secondary reabsorption of water, which leads to an increased plasma volume and vascular response to angiotensin, and consequently an increased systemic vascular resistance (7). In our study, AC patients showed a tendency to have high TFC values, which suggests an important role of high TFC in the process of AH development in this group of patients; however, there are a number of authors who emphasize that a high TFC does not seem to be the key factor in AH development in patients with AC (20). The tendency for fluid retention can be explained as a pathophysiologic phenomenon. Both GH and IGF-1 have antidiuretic effects, which lead to increased plasma volume. If this coincides with other GH- and IGF-1-induced problems (such as overstimulation of the smooth muscle tissue in small blood vessels and the resulting fibrosis, vasoconstriction, insulin resistance, hyperinsulinemia, and excessive sympathetic system activation), the risk of inadequately controlled AH increases considerably (7, 38, 41). Moreover, an increased TFC may be also responsible for subclinical myocardial dysfunction, which seems to be indicated by the results described below.

### Hemodynamic Dysfunction of the Left Ventricle

Our study showed that patients from the AC group have lower cardiac pump function parameters (SI, HI, and ACI) than those in the CG. These findings were also confirmed *via* echocardiographic examinations, with AC patients exhibiting lower LVEF values than CG patients. These findings are consistent with those reported by other authors. Moreover, many study reports emphasize that many patients with AC exhibit structural myocardial remodeling, which is associated with LV hypertrophy and concentric remodeling (14, 24). This is followed by myocardial fibrosis, which is directly associated with GH effects, not only with LV hypertrophy or AH. Cardiac fibrosis impairs LV hemodynamic function, which first manifests in the form of a diastolic dysfunction and later leads to impaired systolic function and the development of symptomatic HF (2, 8, 9, 25, 42, 43). Diastolic LV dysfunction affects approximately 30% of untreated AC patients (41). Importantly, over 60% of adults with AC suffer from AH, and the length of time from AC diagnosis seems to be correlated with AH development. AH, which affects over 60% patients with AC, significantly exacerbates the structural and functional LV dysfunction. One study demonstrated that it was DBP that was the best predictor of LV hypertrophy (8). The observed increases in both CO and CI values during early AC-associated cardiomyopathy and the reduction in SVRI values have also been considered in a search for the possible mechanisms behind AH development in AC (8, 9, 13, 44). Our study showed low values of cardiac pump function parameters (SI and CI) in AC patients, even despite well-controlled AH; this corresponded with echocardiographic evidence of abnormal LV function. Studies in other populations of patients with HF and essential AH have demonstrated a correlation between the values of ICG-assessed hemodynamic parameters and those of echocardiographic indices of LV function (17–19, 45, 46).

### Increase in HR

The results of our study also demonstrated that AC patients were characterized by a significantly higher HR and a slightly higher CI in comparison with those in the CG. These findings are consistent with earlier reports of concentric LV hypertrophy and increased contractility and HR during this early, subclinical, and reversible phase of cardiomyopathy (2, 8, 10). Therefore, hemodynamic abnormalities initially manifest as a hyperkinetic heart, and not until later do they take the form of impaired cardiac pump function (8, 13, 47). Lower creatinine levels in acromegalic patients as compared to controls could be partially explained by renal hypertrophy and consequently enhanced renal filtration process (48).

### Clinical Implications

The study presented here showed that ICG results may be an important indicator of subclinical LV dysfunction in AC patients, even in those with well-controlled AH. Due to its capacity to assess the cardiovascular hemodynamic function in AC patients, ICG seems to be of considerable clinical importance, which is measured *via* the usefulness of this technique in risk stratification in this patient group. It seems particularly important to detect hemodynamic abnormalities in this population before clinical symptoms develop, i.e. at the stage of subclinical disease, when an early therapeutic intervention may prevent fully symptomatic cardiovascular complications. The pathological mechanisms of AH in this patient population are a good argument for initiating antihypertensive combination therapy of vasodilators (angiotensin-converting enzyme [ACE] inhibitors; calcium channel blockers [CCB]) and diuretics.

### Limitations of the Study

The main limitation of the study was the relatively small sample size. This was due to a low prevalence of AC due to a hormonally active pituitary tumor. Many patients with AC had already exhibited comorbidities or evidence of severe cardiovascular dysfunction at the time of diagnosis, and were thus excluded from this study, further diminishing the size of the study population. ICG result interpretation must account for the potential effects of the antihypertensive therapy used i.e. cardiodepressive action of beta-blockers, vascular effect of ACEI/CCB and influence of diuretics on fluid status. However, no significant intergroup differences in pharmacotherapy limits these bias in our study. Likewise, the effect of patient sex on hemodynamic abnormalities in hormone secreting pituitary tumors requires further studies.

## Conclusions

1. The AC-associated hormonal imbalance may lead to cardiovascular dysfunction, which manifests with increased TFC and HR and decreased cardiac contractility indices,
2. Personalized ICG assessments of patients with AC may be useful in detecting early cardiovascular complications and individual hemodynamic profiles, which may help select the optimal combination of antihypertensive drugs, which in turn may improve the prognosis.

## Data Availability Statement

The original contributions presented in the study are included in the article/**Supplementary Material**. Further inquiries can be directed to the corresponding author.

## Ethics Statement

The study was conducted in accordance with the Declaration of Helsinki and the principles of good clinical practice (GCP). The study protocol had been approved by the institutional ethics committee at the Military Institute of Medicine in Warsaw (approval No. 76/WIM/2016). Written informed consent to participate in this study was provided by the participants’ legal guardian/next of kin.

## Author Contributions

Study concept and design, data acquisition and interpretation, and manuscript corrections: AJ, GG, PK, PW, GZ, BU-Ż, AK, and RW. Data analysis and manuscript editing: AJ, PK, GG, PW, and BU-Ż. The final version was approved by all the authors.

## Funding

This study was conducted with the use of the State-allocated research funds granted to the Military Institute of Medicine in Warsaw (WIM/MNiSW grant No. 453/WIM).

## Conflict of Interest

The authors declare that the research was conducted in the absence of any commercial or financial relationships that could be construed as a potential conflict of interest.

## Publisher’s Note

All claims expressed in this article are solely those of the authors and do not necessarily represent those of their affiliated organizations, or those of the publisher, the editors and the reviewers. Any product that may be evaluated in this article, or claim that may be made by its manufacturer, is not guaranteed or endorsed by the publisher.
