# Supplementary material for: Acromegaly: The Research and Practical Value of Noninvasive Hemodynamic Assessments via Impedance Cardiography
Source: Front Endocrinol (Lausanne). 2022 Jan 18;12:793280. doi: 10.3389/fendo.2021.793280 (PMC8805171; doi:10.3389/fendo.2021.793280)
Supplement: Supplementary file 1 [file Table_1.docx]

Supplementary Table S1

*Table S1. Correlations between evaluated chosen hemodynamic parameters and GH/IGF1*

| **Hemodynamic parameter** | **versus IGF-1  (R _Spearman_)** | **versus GH  (R _Spearman_)** |
| --- | --- | --- |
| **HR** | -0.36 | -0.01 |
| **MBP** | -0.24 | -0.31 |
| **PP** | -0.15 | 0.03 |
| **SI** | -0.04 | 0.29 |
| **CI** | -0.26 | 0.34 |
| **SVRI** | 0.12 | -0.34 |
| **TFC** | -0.01 | -0.21 |
| All non-significant (p>0.1)  Abbreviations: CI ‑ cardiac index; HR ‑ heart rate; MBP ‑ mean blood pressure; PP ‑ pulse pressure; SI ‑ stroke index; SVRI ‑ systemic vascular resistance index; TFC ‑ thoracic fluid content | | |
